# A Flexible Hybrid Generator for Efficient Dual Energy Conversion from Raindrops to Electricity

**DOI:** 10.1002/advs.202404310

**Published:** 2024-06-19

**Authors:** Yonghui Zhang, Jiahao Zhang, Huanxi Zheng, Yue Zhao, Yang Chen, Yuyang Zhou, Xiu Liu

**Affiliations:** ^1^ State Key Laboratory of High‐performance Precision Manufacturing Dalian University of Technology Dalian 116024 P. R. China

**Keywords:** droplet‐based electricity generator, efficient energy conversion, electromagnetic generator, flexible hybrid electricity generator, raindrops

## Abstract

Electromagnetic generators are conventionally used to harvest energy from large water bodies, but they are ineffective for harvesting low hydro‐energy, such as raindrops or fogs, due to their bulky, heavy and immovable. Unfortunately, developing new strategies that are lightweight, small, and have high conversion efficiency to convert such low hydro‐energy into electricity remains a challenge. Herein, a flexible droplet‐based hybrid electricity generator (DHEG) consisting of a droplet‐based electricity generator (DEG) and an electromagnetic generator (EMG) is proposed to convert the dual energy of water droplets into electricity simultaneously. The DHEG is assembled by facilely merging DEG and EMG using **conductive elastic multi‐walled carbon nanotubes/polydimethylsiloxane (MWCNTs/PDMS) film**. The MWCNTs/PDMS film can not only serve as a bottom electrode for switching on the DEG, but also as an elastic component for the EMG to vibrate the coil when impacted by water droplets. Activated by a single 58.2 µL droplet falling from a height of 50 cm, the peak voltage, current and power generated by the DHEG are ≈84.6 V, ≈19.85 mA, and ≈595.8 µW, respectively. The energy conversion efficiency of the DHEG is up to ≈13.8%. This flexible hybrid generator may provide a promising strategy for effectively harvesting energy from raindrops.

## Graphical abstract

A flexible hybrid generator (DHEG) consisting of a droplet‐based electricity generator (DEG) and an electromagnetic generator (EMG) is proposed to efficiently convert the dual energy of raindrops into electricity. Both the DEG and EMG parts can be activated simultaneously when impacted by water droplets. Array‐integrated DHEG systems can deliver high power to electronic devices.

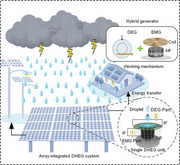

## 1 Introduction

Influenced by global trends such as carbon peaking and sustainable development, technologies for converting renewable natural energy sources into electricity, such as wind, solar, and water, have attracted a great deal of interest.^[^ ^1^, ^2^, ^3^, ^4^ ^]^ As a sustainable source of energy, water, which covers ≈70% of the earth's surface, stores an enormous amount of energy in the form of raindrops, rivers, waves, and fog.^[^ ^5^ ^]^ Electromagnetic generators (EMG) are widely used to produce electricity for large water bodies, but not suitable for relatively low hydro‐energy, such as raindrops or fogs, due to their bulky, heavy, and immovable.^[^ ^6^, ^7^, ^8^ ^]^ Therefore, proposing a reliable strategy for the efficient conversion of these lightweight, low‐volume, and widely distributed water resources remains a great challenge.

Regarding power generation models for harvesting dispersed hydropower, two main approaches, including the surface‐charge method.^[^ ^9^, ^10^, ^11^, ^12^ ^]^ and the electromagnetic induction method based on Faraday's law,^[^ ^13^, ^14^, ^15^ ^]^ have been reported. According to the surface charge method, a number of positive/negative charges at the liquid/solid interface will be carried away.^[^ ^16^ ^]^ Xu et al.^[^ ^17^ ^]^ developed a droplet‐based electricity generator (DEG) that harvested energy from dripping water droplets, which greatly increased the instantaneous power density. Although the method had broken through the limitations of interfacial effects to some extent, the droplet energy conversion efficiency of the DEG was low, ≈2.2%. Based on the method of electromagnetic induction, the excitation component (e.g., spring,^[^ ^18^, ^19^ ^]^ rigid polymer,^[^ ^20^, ^21^, ^22^ ^]^ viscous liquid.^[^ ^23^ ^]^) can be activated by water droplets. The generation of electricity will be generated by changing the magnetic flux of the coil attached to such an assembly. Ma et al.^[^ ^18^ ^]^ proposed a superhydrophobic magnetoelectric generator (SMEG) for converting raindrop energy into electricity with a charge density as high as 51.5 mC m^‐^^2^, which exceeded the electrical performances of currently reported surface‐charge methods. However, the voltage generated by the SMEG was much lower than that of the DEG. Furthermore, raindrop energy harvesting, whether based on the surface‐charge method or the electromagnetic induction method, cannot simultaneously achieve high voltage output and high energy conversion efficiency through a single method.

Recently, a hybrid energy harvester consisting of DEG and C‐S TENG^[^ ^24^ ^]^ has been reported to improve the energy harvesting efficiency from a single droplet, which demonstrates the feasibility of the hybrid system for energy harvesting.^[^ ^25^ ^]^ Although the energy supply performance of the hybrid electricity generator was 25% higher than that of a single DEG, the overall energy conversion efficiency was still low and the device was complex to fabricate. Additionally, a flexible hybrid electricity generator based on EMG and C‐S TENG was proposed to harvest tiny raindrops energy efficiently. Although the EMG part can output a high current, which was attributed to the sensitivity of the elastic film, the generated voltage remained low.^[^ ^26^ ^]^ Among these designs, the movement of the electrical components are limited by the solid medium, and the devices are exposed to moisture, which results in insufficient charge transfer and long‐term stability. Moreover, these hybrid systems are not able to simultaneously output high voltage and high conversion efficiency. Therefore, we speculate that a flexible hybrid system integrated with DEG and EMG may provide an effective solution for outputting both high voltage and energy conversion efficiency from raindrops.

Here we propose a flexible droplet‐based hybrid electricity generator (DHEG) based on hybridization of DEG and EMG with high output voltage and energy conversion efficiency. The DHEG mainly consists of a conductive elastic multi‐walled carbon nanotubes/polydimethylsiloxane (MWCNTS/PDMS) film, two aluminum (Al) electrodes, a fluorinated ethylene propylene (FEP), an NdFeB magnet, an acrylic housing, and a bottom base. By using conductive elastic MWCNTs/PDMS film, the DEG and EMG can be facilely merged. The conductive elastic MWCNTs/PDMS film not only acts as the bottom electrode of the DEG to switch on the circuit when impacted by water droplets but also allows the coil to vibrate steadily, causing a magnetic flux change through the coil. Consequently, electricity can be generated from the dual energy of water droplets by the DHEG. In addition, the use of a closed design and the superhydrophobic film provide a well protection for the internal components. At a single 58.2 µL droplet impact, the peak output voltage, current, and power of the DHEG are ≈84.6 V, ≈19.85 mA, and ≈583.2 µW, respectively. The energy conversion efficiency of the DHEG is up to ≈13.8%. Furthermore, structural and water droplet parameters are used to guide the electrical outputs of the DHEG, and multi‐unit DHEG arrays can serve as a source of power for electronic devices, e.g., LED, capacitor, and clock. This flexible hybrid electricity generator may provide a promising strategy for effectively harvesting energy from dispersed hydropower.

## 2 Results and Discussion

### 2.1 Structure of the DHEG and Performance of the MWCNTs/PDMS Film

**Figure 1:**
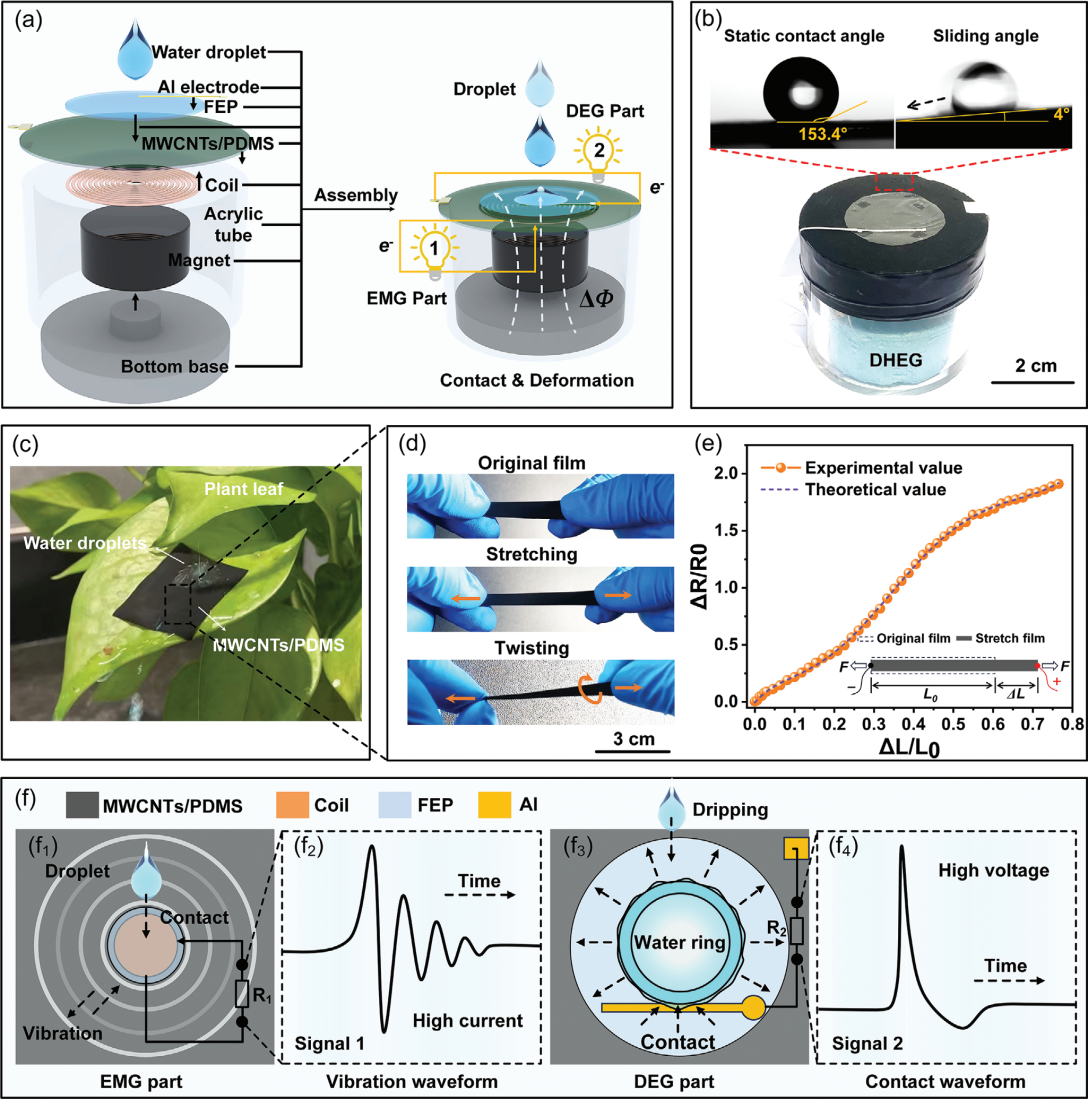
The schematic of the flexible droplet‐based hybrid electricity generator (DHEG) and the performances of the MWCNTs/PDMS film. a) Schematic illustration of the DHEG. b) Optical photographs of the as‐prepared DHEG. Static contact angle and sliding angle of the MWCNTs/PDMS film surface using a 10 µL water droplet. c) Optical image of water droplets impacting the MWCNT/PDMS film placed on the plant leaf. d) Optical images of the MWCNTs/PDMS film when subjected to different extents of linear stretching and twisting, and e) the corresponding conductivity vibration. f) Schematic diagrams of the DHEG power generation, including the (f_1_, f_2_) EMG part and the (f_3_, f_4_) DEG part.

**Figure** **1**a shows a prototype flexible droplet‐based hybrid electricity generator (DHEG), which consists of four main parts from top to bottom: an FEP film with two Al electrodes, an elastic MWCNTs/PDMS film containing a coil, an acrylic housing, and an NdFeB magnet with a bottom base. The FEP film is smooth and hydrophobic with a water contact angle of 101.2°± 0.5° (Figure S1, Supporting Information), which allows for rapid contact and separation of water droplets from the surface. By properly assembling these components together, a DHEG unit (diameter 5 cm, height 4 cm) consisting of two parts (DEG and EMG parts) is created, which can efficiently convert the dual energy of water droplets into electricity simultaneously (Figure 1b). Furthermore, the device is completely enclosed, which effectively protects the internal components of the DHEG. Here, an elastic MWCNTs/PDMS film, a coil with an internal resistance of 13.2 Ω, and a NdFeB magnet yielding a surface magnetic intensity of ≈421 mT are employed.

The conductive elastic MWCNTs/PDMS film that function as a main structure of the DHEG not only receives excitations from droplets but also protects the inner components from water or vapor erosion. The film is fabricated by a simple and environmentally friendly method, including spin‐coating, powder‐coating, curing, and peeling off (Figure S2, Supporting Information). After the coating process, the sheet‐like conductive MWCNTs layer is successfully adhered to the PDMS surface (Figure S3, Supporting Information), which is superhydrophobic and allows water droplets to roll off the surface quickly, with a static water contact angle (WCA) of ≈153.4° and a rolling angle (RA) of ≈4°, as shown in Figure 1b. Due to the strong bonding of the micro‐ and nanostructures on the surfaces, the elastic MWCNTs/PDMS film retains its superhydrophobic properties despite multiple abrasions (Video S1, Supporting Information). Besides, the elastic film can recover quickly from repeated stretching and twisting and maintain a stable resistance vibration (Figure 1d,e; Video S1, Supporting Information). Therefore, it is reasonable to believe that the film can be well used in the DHEG for power generation in harsh environments.

Based on Faraday's law of electromagnetism (EMG part) and the surface charge method (DEG part), the DHEG can convert the dual energy of the water droplets into electricity simultaneously, as shown in Figure 1f. On the one hand, due to the elasticity of the film, the coil can continue to vibrate after the droplets impacted the DHEG surface, causing a change of magnetic flux through the coil, which generated an induced electromotive force with high current (Figure 1f_1_,f_2_). On the other hand, when falling droplets spread and contact the upper Al electrode on the surface of the DEG, the original open circuit can be switched on, allowing an instantaneous high voltage to be generated (Figure 1f_3_,f_4_). Consequently, the dual energy (including mechanical and electrostatic energy) of the droplets can be successfully converted into electricity by a DHEG unit.

### 2.2 Working Mechanism and Electrical Output of the DHEG

The DEG and EMG of the DHEG can simultaneously generate electrical output when activated by a single water droplet. In the experiment, the surface charges of the dielectrics of the DEG part are assumed to be completely saturated by successive droplet dripping operation on the DHESFG based on the reported method.^[^ ^27^, ^28^ ^]^ The power generation principle of the DHEG can be illustrated as follows:

In the DEG part, the surface charges of the dielectric (FEP) and the induced charges in the lower electrode (the conductive MWCNTs layer) keep an electrostatic equilibrium before droplet impact (Figure S4a(Ι), Supporting Information). A water droplet falling from a height impacts the DHEG and spreads over the surface of FEP to form a film‐like water ring (Figure 1f_3_). Part of the kinetic energy of the droplet can be converted into the surface energy of the interface between the dielectric and the water layer. During the spreading process, the droplet gradually approaches and contacts the upper Al electrode, and the ions and dipoles stored in the droplet will be redistributed by the electrostatic force.^[^ ^18^ ^]^ Attracted by the negative charges on the surface of the dielectric, the positive ions in the droplet are aligned near the dielectric, while the negative ions in the droplet tend to move away. Due to the screening effect of positive ions and surface charges,^[^ ^25^ ^]^ part of the induced charges in the lower electrode (the conductive MWCNTs layer) are diverted to the upper Al electrode by the electrostatic induction, thus generating a current (Figure S4a(ΙΙ,ΙΙΙ), Supporting Information). As the surface tension is reduced after the droplet spreads to its maximum, the screening effect is weakened, resulting in a remigration of the induced charges between the upper and lower electrodes, i.e., a reverse current can be produced (Figure S4a(ΙV), Supporting Information).

In the EMG part, the gap between the coil and the magnet remains constant prior to droplet impact (Figure S4b(Ι), Supporting Information). The impact of the droplet deformed the elastic MWCNTs/PDMS film, driving the coil downward, causing a magnetic flux change through the coil, generating an induced electric potential (Figure S4b(ΙΙ), Supporting Information). When the kinetic energy of the droplet is less than the restoring energy of the film, the coil moves upward as the film rebounds, generating the opposite induced electromotive force (Figure S4b(ΙΙΙ), Supporting Information). When the coil moves to the highest point, the droplet will leave the surface of the DHEG (Figure S4b(ΙV), Supporting Information). Finally, the magnetic flux through the coil shows an oscillatory decay after the droplet leaves the surface of the DHEG.

**Figure 2:**
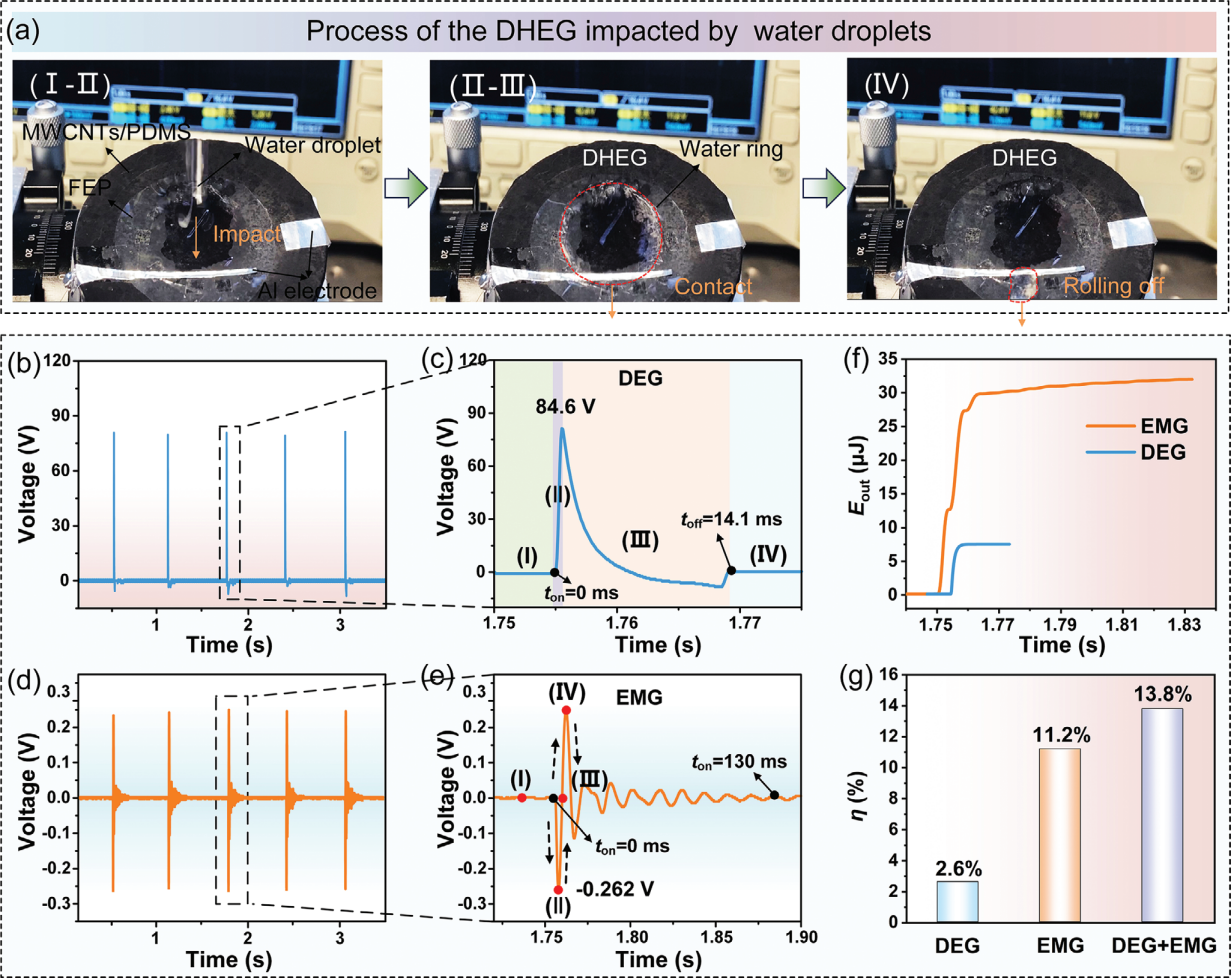
Effect of water droplets on the DHEG and its electrical outputs. a) The process of the DHEG is impacted by water droplets. The output voltage of b,c) the DEG and d,e) EMG parts under continuous water droplet impacts. f) Comparison of the amount of electrical output energy (*E*_out_) of the DEG and the EMG, and g) the corresponding raindrop energy conversion efficiencies (*η*). Here, 58.2 µL water droplets dropped from a height of 50 cm are used in the test.

Based on the power generation principle of the DHEG, the electrical signal can be successfully generated by a single water droplet impact. When the DHEG is impacted by continuous 58.2 µL water droplets, the peak voltages generated by the DEG and EMG parts are as high as ≈84.6  and ≈0.262 V, respectively (**Figure** **2**b–e), and corresponding peak currents are ≈84.6 µA and ≈19.85 mA, respectively (Figure S5a,b, Supporting Information). The charges yielded by the DEG and EMG parts are ≈7.07 µC and ≈2.48 C, respectively (Figure S5c, Supporting Information), which can be calculated as the charge density of 17.33 mC m^−2^ (the maximum spreading area of water droplets is ca. 4.08 cm^2^) and 1631.84 C m^−2^ (the top surface of EMG is ca. 15.2 cm^2^), respectively. Therefore, the total charge density of a DHEG unit is ≈1631.842 C m^‐2^. Compared with the DEG, the EMG can generate a lower voltage but a higher current, which can be mainly attributed to the fact that the internal resistance of the DEG is much larger than that of the EMG. Besides, the electrical energy converted by the EMG part is much higher than that of the DEG. The generated electrical energies of the DEG and EMG parts are ≈7.421 µJ and ≈31.96 µJ, respectively (Figure 2f), and the corresponding conversion efficiencies generated are up to ≈2.6% and ≈11.2%, respectively (Figure 2g). An important reason is that EMG can generate electricity last longer than DEG, ≈9.2 times longer (Figure 2c,e). The total conversion efficiency of the DHEG is ≈13.8% (Figure 2g), which is 5.3 times higher than that of a single DEG and is beyond the efficiency converted by the reported studies (Table S1, Supporting Information).

### 2.3 Working Circuit, Simulation, and Calculation of the DHEG

When a water droplet impacts the DHEG, the DEG, and EMG can generate electricity independently of each other, with the DEG generating power based on the surface charge method and the EMG generating power using Faraday's law of electromagnetic induction. As shown in Figure 2a, a water droplet impacting the DHEG can switch on the circuit of the DEG and at the same time cause the coil to vibrate. Based on the previous analysis, it can be seen that the power generation time of the DEG is slightly lagging behind that of the EMG, and the energy conversion efficiency is also lower than that of the EMG (Figure 2b–g). To further investigate the working mechanism of the DHEG, the following analyses are conducted.

On the one hand, an equivalent circuit model is used to analyze the output behavior of the DEG, as shown in Figure S6a (Supporting Information). Here, the water droplet can be considered a resistor (*R*_w_).^[^ ^29^ ^]^ First, the entire circuit is disconnected when the water droplet impacts the dielectric (FEP) surface before contacting the upper Al electrode, and an electric double layer.^[^ ^30^ ^]^ with a capacitance of *C*_1_ is created at the water/dielectric interface. Another capacitance formed at the dielectric/ the lower electrode (the conductive MWCNTs layer) interface is treated as *C*_2_. Second, as the water droplet spread and contacted the upper Al electrode, a new capacitance (*C*_3_) is created in the double layer between the water and the upper Al electrode, thus connecting the circuit. Compared to the disconnected state, the overall equivalent capacitance *C* is significantly reduced, which can be calculated by Equation (1).

(1) $$\frac{1}{C}=\frac{1}{C_{1}}\;+\frac{1}{C_{2}}+\;\frac{1}{C_{3}}=\frac{d}{\varepsilon \cdot S_{1}}\;+\frac{d}{\varepsilon \cdot S_{2}}+\frac{d}{\varepsilon \cdot S_{3}}$$

Where *S*_1_, *S*_2_, *S*_3_, ε, and *d*, are the maximum spreading area of the water droplet, the contact area between the dielectric (FEP) and the lower electrode (the conductive MWCNTs layer), an instant contact area between the water and the upper Al electrode, dielectric constant, and the thickness of electric double layer, respectively.

*C*_3_ is formed at the instant contact between the water and the upper Al electrode, the contact area *S*_3_ is extremely small and much smaller than *S*_1_ and *S*_2_ (i.e., *S*_2_ > *S*_1_ ≫ *S*_3_). Therefore, it can be assumed that *C* is approximately equal to the smaller *C*_3_ (i.e., *C*≈*C*_3_). Accordingly, the output voltage (*V*) of the DEG can be calculated by the following equation (Equation (2).^[^ ^31^ ^]^:

(2) $$V\;=\frac{Q}{C}\;\approx \;\frac{Q}{C_{3}}=\frac{\sigma \cdot s_{1}}{\frac{\varepsilon \cdot s_{3}}{d}}\;$$

where *Q* is the charges induced at the water/dielectric interface when impacted by the water droplet, *σ* is the surface charge density of the solid surface.^[^ ^32^ ^]^

Finally, as the contact area between the water droplet and the upper Al electrode increases, the capacitance *C* increases, leading to a decrease in the voltage. When the contact area reaches maximum, the voltage gets close to 0 V. Furthermore, when the contact area decreases, the capacitance decreases, causing a reverse electrical response. However, the surface charge of the solid surface is taken away by the water droplets, making the reverse voltage lower than the forward voltage. The above analysis is consistent with the experimental results (Figure 2b,c).

On the other hand, the excitation of the DHEG by the water droplet can induce an electrical response in the EMG. Based on Faraday's laws of electromagnetism, an electrical response can be generated in the EMG part when a magnetic flux change occurs in the coil. As illustrated in Figure S6b (Supporting Information), the flux changes increase when the coil gets close to the magnet and decrease vice versa.

The corresponding curve of magnetic field intensity versus gap is shown in Figure S7 (Supporting Information). The short‐circuit current (*I*_sc_) induced in the EMG part can be calculated using Equation (3).^[^ ^23^, ^33^ ^]^:

(3) $$I_{\mathrm{SC}}=-N\;\frac{\Delta \Phi }{\Delta t\cdot R_{\mathrm{coil}}}=-N\frac{\Phi_{\mathrm{after}}-\Phi_{\mathrm{before}}}{\Delta t\cdot R_{\mathrm{coil}}}$$

Where *N*, *R*_coil_, ΔΦ, and Δ*t* are coil turns, coil resistance, the change in the total magnetic flux through the coil, and the time at which the water droplet impacts the EMG, respectively.

Besides, the DEG and the EMG constitute the DHEG, and thus the energy‐conversion efficiency of the DHEG can be calculated by Equation (4):

(4) $$\begin{array}{c} \eta =\eta_{\mathrm{DEG}}+\eta_{\mathrm{EMG}}=\frac{E_{\mathrm{DEG}\;+}E_{\mathrm{EMG}}}{E_{\mathrm{Droplet}}}=\frac{\int_{t_{\mathrm{on}}}^{t_{\mathrm{off}}}\frac{{U_{\mathrm{DEG}}}^{2}}{R_{E}}dt+\int_{t_{\mathrm{on}}}^{t_{\mathrm{off}}}\frac{{U_{\mathrm{EMG}}}^{2}}{R_{\mathrm{coil}}}dt}{\mathrm{mgh}} \end{array}$$

where the droplet mass, *m*, is 0.0582 g; the gravitational acceleration, *g*, is 9.8 m s^2^; the drop height, *h*, is 50 cm; the impedance of the external loads, *R*_E_ and *R*_coil_, are 1 MΩ and 13.2 Ω, respectively; *t*_on_ and *t*_off_ are the onset and termination times of the electrical signals generated by the DEG and EMG parts, respectively, when a single water droplet impacts on the DHEG. *U*_DEG _and *U*_EMG _are the voltages generated by the DEG and EMG parts, respectively; *E*_DEG _and *E*_EMG _are the harvested energy generated by the DEG and EMG parts, respectively. *E*_Droplet _is the kinetic energy carried by a water droplet falling from a height; *η*, *η*_DEG_ and *η*_EMG_ are the total conversion efficiency, the conversion efficiency of the DEG and the EMG, respectively.

### 2.4 Effect of Structural and Water Droplet Parameters on the Electrical Output Performance of DHEG

**Figure 3:**
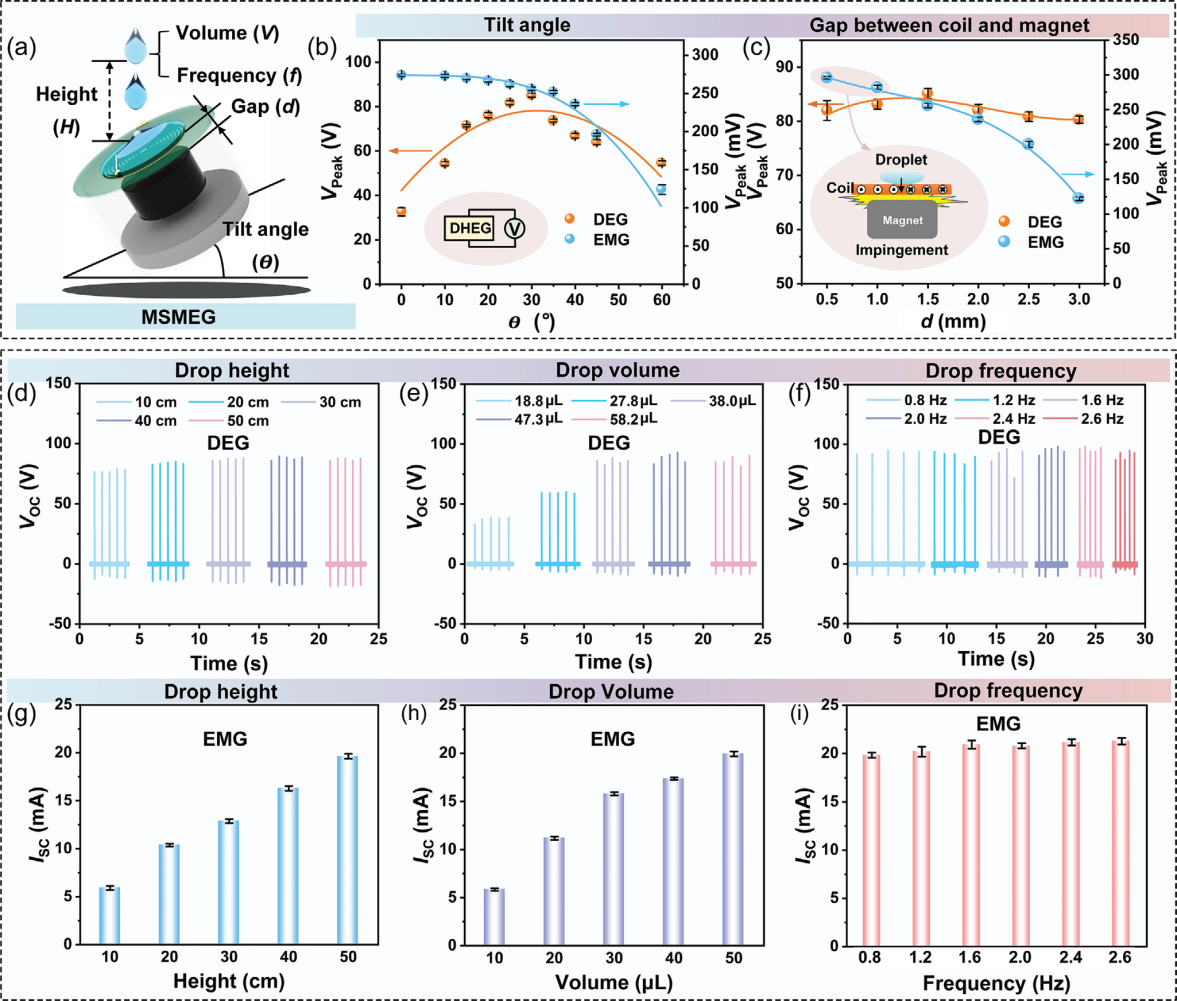
Effect of structural and water droplet parameters on electricity produced by the DHEG. a) Schematic illustration of different parameters affecting the output performance of the DHEG. b,c) *V*_OC_ for the DEG and EMG parts at different tilt angles (𝜭) and gaps (*d*). d) *V*_OC_ of DEG and g) *I*_SC_ of EMG at different drop heights (*H*). e) *V*_OC_ of DEG and h) *I*_SC_ of EMG at different drop volumes (*V*). f) *V*_OC_ of DEG and i) *I*_SC_ of EMG at different drop frequencies (*f*). Here, the drop volume, height, frequency, and tilt angle used in the test are 58.2 µL, 50 cm, 2 Hz, and 30°, respectively, except where noted.

Given that the DHEG structure may affect the electricity output performance, the gap (*d*) between the coil and magnet and the tilt angle (𝜭) are considered as two main parameters to be evaluated, as shown in **Figure** **3**a–c. As the tilt angle increases, the open circuit voltage (*V*_OC_) of the DEG increases and then decreases, reaching a peak *V*_OC_ of ≈84.6 V at 𝜭 = 30°. For the EMG, the *V*_OC_ presents a decreasing trend (Figure 3b). Furthermore, the output performance of the DHEG is investigated at different gaps based on 𝜭 = 30°, as shown in Figure 3c. In the case of the DEG, no significant difference exists between the voltage outputs, and *V*_OC_ reaches a maximum value of ≈86.4 V at *d *= 1.5 mm. Although higher voltages can be generated in the EMG part at *d*<1.5 mm, the gap is so small that the coil will hit the bottom magnet (Figure 3c), resulting in a lower charge. Therefore, tilt angle 𝜭 = 30° and gap *d *= 1.5 mm will be selected as the optimal structural parameters to investigate the effect of different droplet parameters on the output performance of the DHEG.

To investigate the effect of water droplet parameters on the electrical performance of the DHEG, the *V*_OC_, and *I*_SC_ of the DEG and EMG parts are tested under different conditions, including drop height (*H*), volume (*V*), and frequency (*f*), as shown in Figure 3d–i. For the DEG part, the *V*_OC_ changes slightly as the drop height rises from 10  to 50 cm (Figure 3d), but the *I*_SC_ increases gradually and obtains a maximum value (≈87.9 µA) at *H *= 40 cm (Figure S8a,b, Supporting Information). As seen in Figure 3f and Figure S8e,f (Supporting Information), the *V*_OC_ and *I*_SC_ obtained at different drop frequencies exhibit a similar flat trend, which is consistent with previous reports and attributed to a constant load. However, both *V*_OC_ and *I*_SC_ first rise with increasing drop volume and then stay at ≈85.6 V and ≈86 µA at *V *> 38 µL (Figure 3e; Figure S8c,d, Supporting Information). For the EMG part, the *V*_OC_ and *I*_SC_ increase with the drop height and volume, reaching the maximum *V*_OC_ and *I*_SC_ of ≈263.2 mV and ≈19.2 mA at *H *= 50 cm and *V *= 58.2 µL (Figure 3g,h; Figure S8, Supporting Information), respectively. Besides, the electrical outputs of the EMG show a similar trend to that of the DEG at different drop frequencies, with a current of ≈20.4 mA (Figure 3i; Figure S8, Supporting Information).

### 2.5 Power Generation and Applications

**Figure 4:**
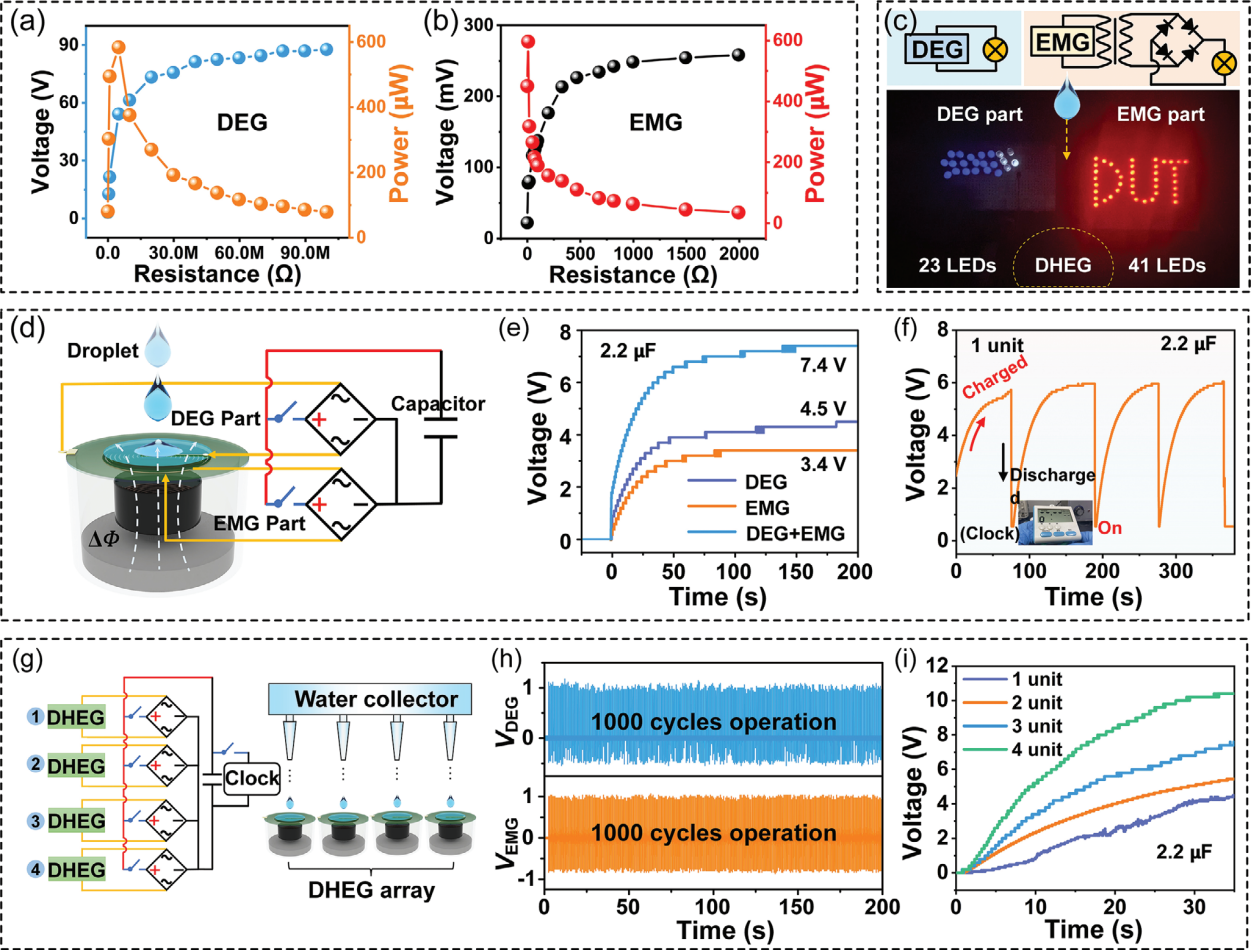
Electrical output performance of the DHEG and its application. a,b)Electrical outputs of the DEG and EMG parts under different external loads, where the DHEG is driven by a single water droplet (volume 58.2 µL, drop height 50 cm, drop frequency 2 Hz). c) Optical images of continuous droplets impacting the DHEG to light up LEDs. The built‐in circuit diagrams show the schematic of the connection circuits for the DEG and EMG parts, respectively. d) Schematic diagram of a single DHEG charging a capacitor. e) Voltage diagram for charging a 2.2 µF capacitor by different generating parts, including DEG, EMG, and DHEG. f) Voltage plot for charging a 2.2 µF capacitor to drive a clock by successive single‐droplet impacts on a single DHEG unit. g) Optical image of DHEG array charging capacitor and g) the corresponding circuit. h) Normalized voltage outputs of the DEG and EMG parts for 200 s. i) Voltage plot for charging a 2.2 µF capacitor by a different number of DHEG units.

Although the DHEG can be activated by a single water droplet, its electrical output performance determines whether it can be used in portable devices. Both DEG and EMG can output a stable electrical response under continuous water droplet impact, which indicates that the electrical output performance of DHEG can be evaluated by connecting different external loads. As shown in **Figure** **4**a,b, different external resistors are connected in series with the DEG and EMG parts, and their peak voltage, current, and power are tested. The output voltages of both the DEG and the EMG increased with load resistance, while the corresponding currents show a decreasing trend in the low region, with peak power of ≈583.2 µW (equivalent to the power density of 1.18W m^‐^^2^) and ≈595.8 µW (equivalent to the power density of 392.09 mW m^‐^^2^) in the DEG part and the EMG part, respectively, at the load resistances of ≈5 MΩ and 10 Ω. Therefore, the total power density of a DHEG unit was ≈1.572 W m^‐^^2^. The electrical output performance of the DHEG and different raindrop‐based energy harvesters are summarized in Table S1 (Supporting Information), showing that the DHEG can output higher current and charge density.

Based on the maximum power transferred from the DHEG to the external resistance, the DHEG can be used as a power supply for electronic devices. LEDs lighting, capacitor charging, and clock driving confirm the viability of the DHEG as a power source (Figure 4). Driven by a single water droplet, the energy harvested by the DEG from the droplet can illuminate 23 LEDs, while the power generated by the EMG can illuminate an LED array of “DUT” with 41 LEDs, as illustrated in Figure 4c and Video S2 (Supporting Information). Circuits for the DEG and EMG parts to light up are shown in Figure S9, Supporting Information. Additionally, the charging behaviors of the DEG, the EMG, and the DHEG on a capacitor (2.2 µF) are compared to demonstrate a higher charging efficiency of the DHEG (Figure 4d,e). The DHEG can charge the capacitor up to 7.4 V, showing ≈64.4% voltage improvement over a single DEG and ≈117.6% improvement over a single EMG, as shown in Figure 4e. Furthermore, the charging rate decreases as the capacitance increases, and the DHEG can charge the capacitor to saturation within a short time (Figure S10, Supporting Information). The above results indicate that the DHEG has an outstanding electrical output performance. In the case of a single DHEG unit, the current converted by the mechanical energy of the falling droplets can be rectified and charged into the capacitor (2.2 µF). Thus, the charged capacitor can drive the clock under continuous water droplet impacts, as shown in Figure 4f.

Besides, the DHEG can rapidly harvest raindrop energy to power electronic devices by integrating different numbers of DHEG units. Here, each DHEG unit is connected in parallel, and the circuit schematic for fast charging the capacitor is shown in Figure 4g and Figure S11(Supporting Information). The continuous water droplets with a volume of 58.2 µL are released statically from a height of 50 cm using a syringe pump to simulate raindrops impacting the DHEG. In practice, the durability of the DHEG must be given priority. To verify its durability, the DHEG is impacted by continuous water droplets(58.2 µL per drop) for 200 s. Figure 4h displays the normalized voltage outputs of the DEG and EMG parts for 200 s, respectively. Both the DEG and EMG parts can output an adequately stable electric response over long periods of continuous water droplet impacts, and no significant mechanical damage or performance degradation is observed. In addition, the DHEG unit maintains excellent electrical output stability regardless of strong winds and heavy rain (Figure S13 and Video S3, Supporting Information). Here, the system is well sealed and the upper Al electrode is extended to harvest more raindrop energy (Figure S12, Supporting Information). Furthermore, the charge behavior for the capacitor connected with different numbers of DHEG units demonstrates that as the number increases, the capacitor charging rate also increases (Figure 4i). Therefore, multi‐unit DHEG arrays(DHEGs) can be used to rapidly power electronic devices in a rainfall environment.

## 3 Conclusion

In this work, we propose a flexible droplet‐based hybrid electricity generator (DHEG) consisting of a DEG and an EMG to efficiently convert the dual energy of water droplets to electricity. The DEG and EMG parts are easily hybridized into such an energy harvester using an elastic MWCNTs/PDMS film, which can be fabricated in a simple power‐coating operation. The conductive elastic MWCNTs/PDMS film not only acts as a lower electrode for the DEG but also as an excitation component for the EMG, enabling the DHEG to generate a high voltage and current simultaneously. The energy conversion efficiency of the DHEG is up to ≈13.8%, which is much greater than that of a single DEG. Furthermore, the DHEG charges the capacitor faster than that of a single DEG or EMG. Based on the DHEG outstanding output performance, the DHEG as a power source can be successfully used to illuminate LED arrays and charge capacitors to drive electronic devices. By proposing such a hybrid design, this study provides a new frontier strategy in energy harvesting system that efficiently converts raindrop energy into electricity.

## 4 Experimental Section

### Preparation of the Elastic MWCNTs/PDMS Film

First, a mixture of polydimethylsiloxane (PDMS, Sylgard 184, Dow Corning, USA) with a curing agent ratio of 10:1.5 was magnetically stirred for 15 min, followed by vacuum degassing to remove air bubbles. Second, 1 g of the mixtures was spin‐coated on a 5 × 5 cm^2^ glass substrate using a spin‐coating device (KW‐4A, SETCAS Electronics Co., Ltd, China) at 1500 rpm for 20 s. The PDMS‐coated glass substrate was pre‐treated for 2 min at 60 °C by placing it on a thermostatic heater (JF‐956A, Jinfeng Electronic Tools Factory, Changan, Dongguan, China). Third, multi‐walled carbon nanotube powders (MWCNTs, 95% purity, Tanfeng Tech.lnc., China) were uniformly sprayed onto the prefabricated PDMS, and then powder‐coated with a flexible brush for 3 min. Finally, the elastic conductive MWCNTs/PDMS film was obtained by drying at 110 °C for 3 h.

### Assembly of the DHEG

The DHEG consisted of six parts: an elastic MWCNTs/PDMS film, two Al electrodes, a fluorinated ethylene propylene (FEP) film, an NdFeB magnet, an acrylic housing, and a bottom base. The as‐prepared MWCNTs/PDMS film was flattened, tautened, and secured to the top of the hollow acrylic tube (inner diameter 44 mm, outer diameter 50 mm, height 40 mm) by adhesive tape. A FEP film with a thickness of 15 µm and a diameter of 30 mm was attached on the top of the MWCNTs/PDMS film. The top aluminum (Al) electrode with a size of 12 × 1 × 0.06 mm^3^ was applied to the FEP surface with a midpoint offset of 10 mm. Side Al electrode with a size of 10 × 5 × 0.06 mm^3^) was attached to the surface of MWCNTs/PDMS film. A copper coil (internal resistance 13.2 Ω, diameter 2.0 cm, wire diameter 50 µm, thickness 0.2 mm) was glued to the bottom of MWCNTs/PDMS film. The cylindrical NdFeB magnet (N45H, Beijing Jiujiugaoke Magnetic Material Co., Ltd, China) with a diameter of 16.5 mm and a height of 9.2 mm was assembled on the bottom base, and the gap between the top of the magnet and the bottom of the MWCNTs/PDMS film was set by the experiment. The unit was completely sealed when fully assembled.

### Characterization and Measurements

The static water contact angle (WCA) and rolling angle (RA) on the elastic MWCNTs/PDMS film were measured on a contact angle meter (SL200KS, KINO, USA). Scanning electron microscopy of the surface morphology of the MWCNTs/PDMS film was conducted on a tungsten filament scanning electron microscope (SEM, JSM‐6360LV, Japan) at an accelerating voltage of 20 kV. The surface magnetic intensity of NdFeB magnets was detected using a commercial Teslameter (WT10A, WEITE Magnetic Technology Co., Ltd, China). The internal resistances of MWCNTs/PDMS film and coil were measured by a digital multimeter (XDM1041, Fujian Lilliput Optoelectronics Technology Co., Ltd, China). Water droplets were continuously pumped into the dropper using an auto‐injector (LSP02‐2A, Longer pump, China) to impact the DHEG from different heights. The electrical signals of the DHEG under water droplets impact were performed using a digital phosphor oscilloscope (DPO 2014B, Tektronix, America). The magnetic field distribution in the EMG part was simulated using Ansys Electronics.

## Conflict of Interest

The authors declare no conflict of interest.

## Supporting information

Supporting Information

Supplemental Video 1

Supplemental Video 2

Supplemental Video 3

## Data Availability

The data that support the findings of this study are available in the supplementary material of this article.
