# Correction to “A Flexible Hybrid Generator for Efficient Dual Energy Conversion from Raindrops to Electricity”

**DOI:** 10.1002/advs.202502344

**Published:** 2025-02-20

Zhang Y, Zhang J, Zheng H, Zhao Y, Chen Y, Zhou Y, and Liu X. A Flexible Hybrid Generator for Efficient Dual Energy Conversion from Raindrops to Electricity. *Adv. Sci*. **2024**, 2404310.

https://doi.org/10.1002/advs.202404310

The name of the corresponding author “Xiu Liu” was incorrect. This should have read: “Xin Liu”

We apologize for this error.

