# Supplementary material for: A Flexible Hybrid Generator for Efficient Dual Energy Conversion from Raindrops to Electricity
Source: Adv Sci (Weinh). 2024 Jun 19;11(31):2404310. doi: 10.1002/advs.202404310 (PMC11336931; doi:10.1002/advs.202404310)
Supplement: Supplementary file 1 — Supporting Information [file ADVS-11-2404310-s001.docx]

Supplementary Information for

**A Flexible Hybrid Generator for Efficient Dual Energy Conversion from Raindrops to Electricity**

*Yonghui Zhang*, *Jiahao Zhang*, *Huanxi Zheng*, *Yue Zhao*, *Yang Chen*, *Yuyang Zhou*, *Xiu Liu*^*^

Y. Zhang, J. Zhang, Prof. H. Zheng, Y. Zhao, Y. Chen, Y. Zhou, Prof. X. Liu

State Key Laboratory of High-performance Precision Manufacturing, Dalian University of Technology, Dalian 116024, P. R. China

E-mail: [xinliu@dlut.edu.cn](mailto:xinliu@dlut.edu.cn)

**Content**

**Figure S1.** Contact angle between the water droplet (~6 μL) and the FEP film.

**Figure S2.** Schematic diagram of the preparation of the conductive elastic MWCNT/PDMS film.

**Figure S3.** (a) Optical image and (b,c) SEM images of the surfaces of the MWCNTs/PDMS film.

**Figure S4.** Mechanism and output performance of the DHEG based on a single droplet excitation. Operation mechanism of a) the DEG and b) the EMG.

**Figure S5.** a) Voltage measurement circuit for DHEG. b) The output peak currents and c) charges generated from a single droplet impacting on the DEG part and the EMG part, respectively.

**Figure S6.** a) The equivalent circuit model of the DEG part. Only in the connected state, *C*_3_ presents in the closed circuit. *C*_1_, *C*_2_ and *C*_3_ are the capacitor of the electric double layer between the water and the dielectric, the dielectric/bottom electrode interface and the water/upper electrode interface, respectively. *R*_w_, *R*_E_ and *R*_F_ are the impedance of the water droplet, the external load and the surface of the MWCNTs/PDMS film, respectively. b) The magnetic field intensity distributions of the EMG when subjected to the impact of a single water droplet.

**Figure S7.** a) Magnetic field strength (B) distribution of the device and the corresponding measurement point. b) Curve of magnetic field intensity vs. gap.

**Figure S8.** Effect of raindrop parameters on electricity produced by the DHEG. a) *I*_SC_ and *V*_OC_ generated by the DEG and EMG parts, respectively, at different drop heights. b) Schematic illustration of different drop heights impacting the DHEG. c) *I*_SC_ and *V*_OC_ generated by the DEG and EMG parts, respectively, at different drop volumes. d) Schematic illustration of different drop volumes impacting the DHEG. e) *I*_SC_ and *V*_OC_ generated by the DEG and EMG parts, respectively, at different drop frequencies. f) Schematic illustration of different drop frequencies impacting the DHEG. Here, the drop volume, height, frequency and tilt angle used in the test were 58.2 µL, 50 cm, 2 Hz and 30°, respectively, except where noted.

**Figure S9.** (a) Circuit for DEG to light up series-connected LEDs. (b) Circuit for voltage-amplified EMG to light up parallel connected LEDs.

**Figure S10.** Voltage plots of the DHEG (DEG+EMG) for charging different capacitors.

**Figure S11.** Optical image of DHEG array charging capacitor and driving clock.

**Figure S12.** A strategy to improve DHEG for collecting more raindrop energy. A prototype of (a) the original DHEG and (b,c) the improved DHEG.

**Figure S13.** The durability of the system in (a) strong wind and (b) heavy rain environments.

**Table S1.** Comparison of the performance of different raindrop-based energy harvesters reported recently.

**Video S1.** Mechanical stability of the elastic MWCNTs/PDMS film.

**Video S2.** Driven by a single water droplet, the energy extracted by the DEG from the droplet could illuminate 23 LEDs, while the power generated by the EMG could illuminate a LED array of “DUT” with 41 LEDs.

**Video S3.** Durability of the hybrid energy harvesting system.


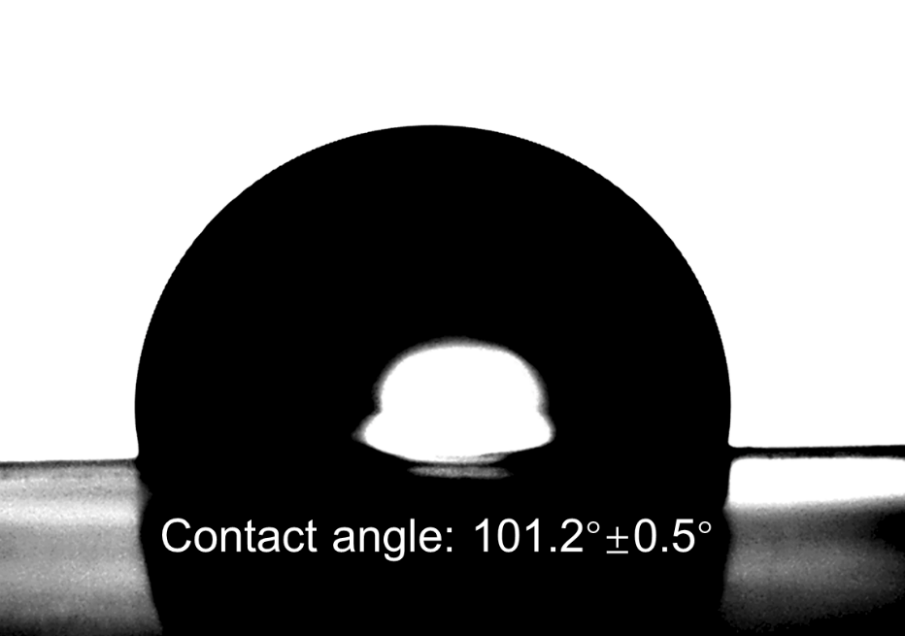


**Figure S1.** Contact angle between the water droplet (~6 μL) and the FEP film.


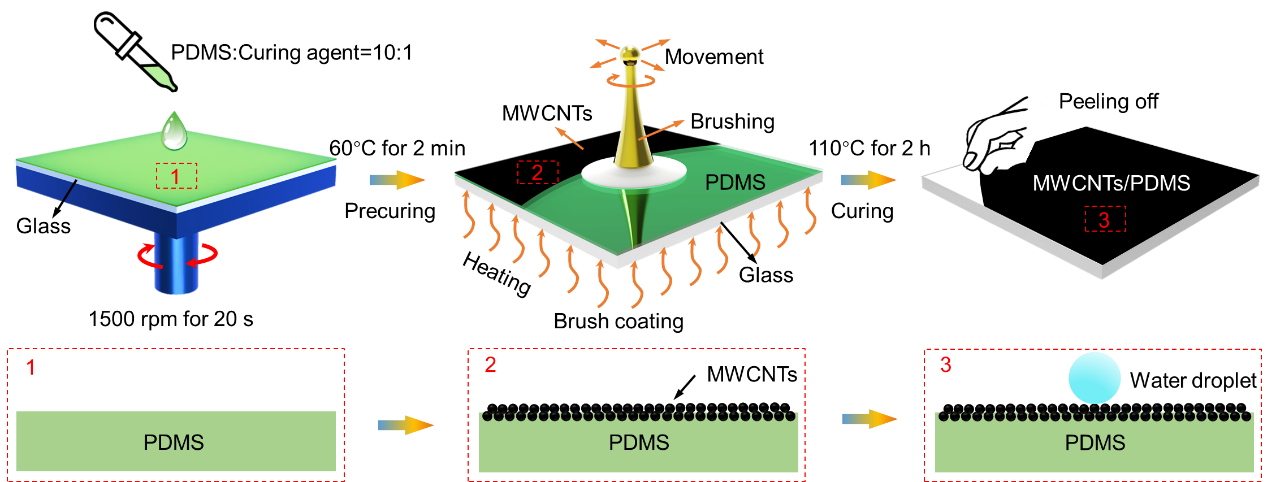


**Figure S2.** Schematic diagram of the preparation of the conductive elastic MWCNT/PDMS film.


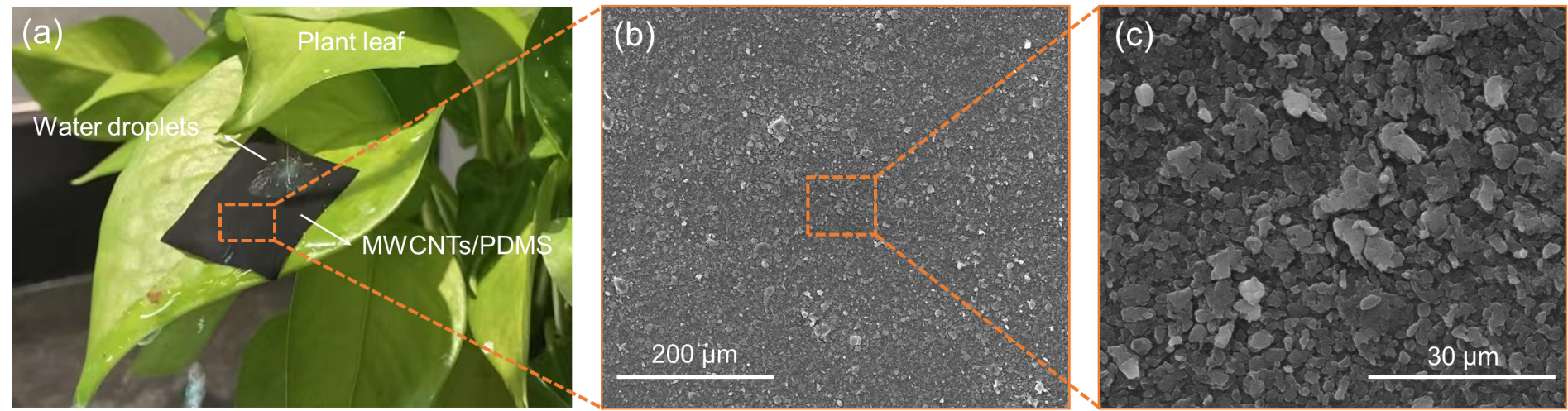


**Figure S3.** (a) Optical image and (b,c) SEM images of the surfaces of the MWCNTs/PDMS film.


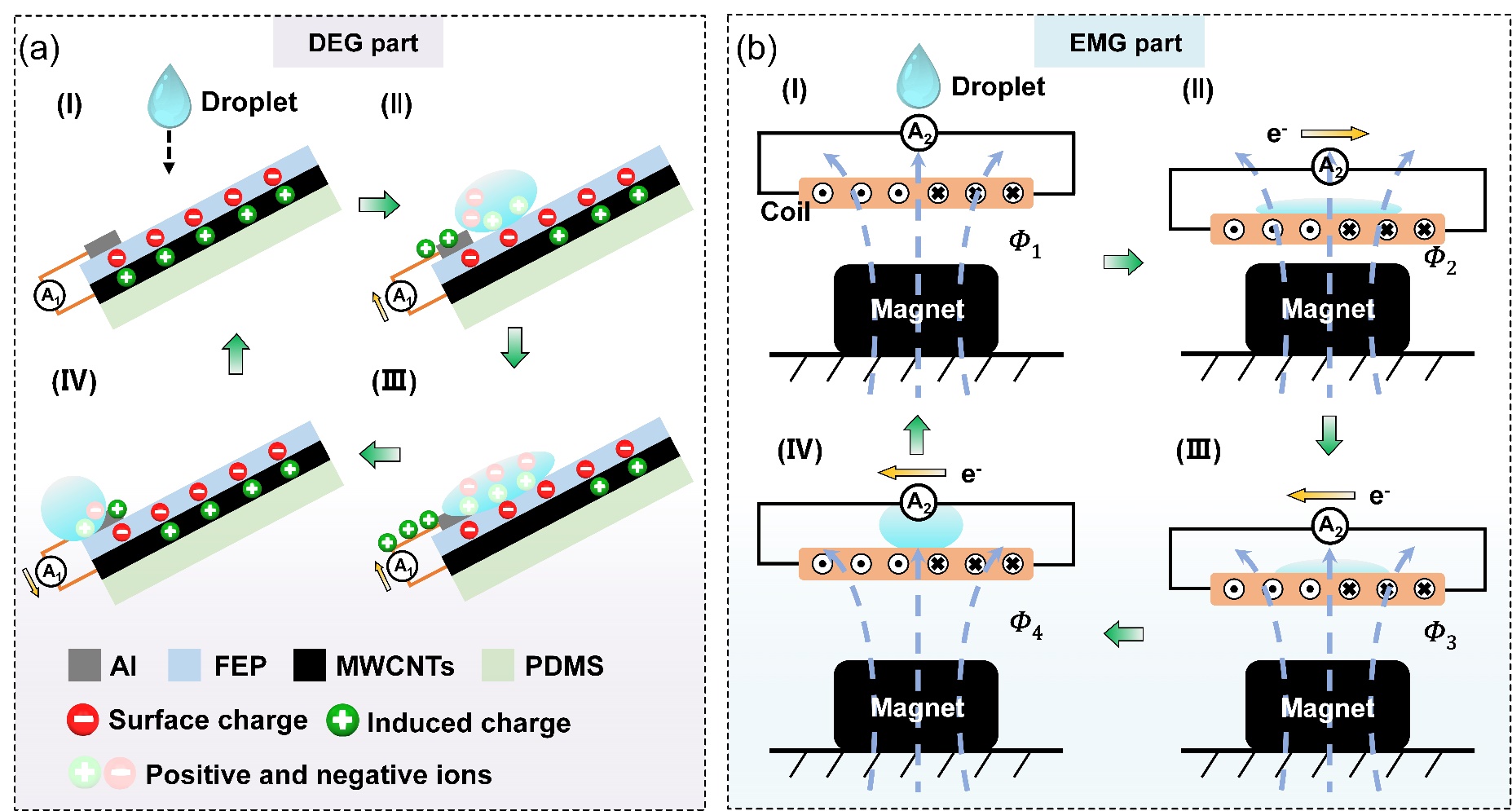


**Figure S4.** Mechanism and output performance of the DHEG based on a single droplet excitation. Operation mechanism of a) the DEG and b) the EMG.


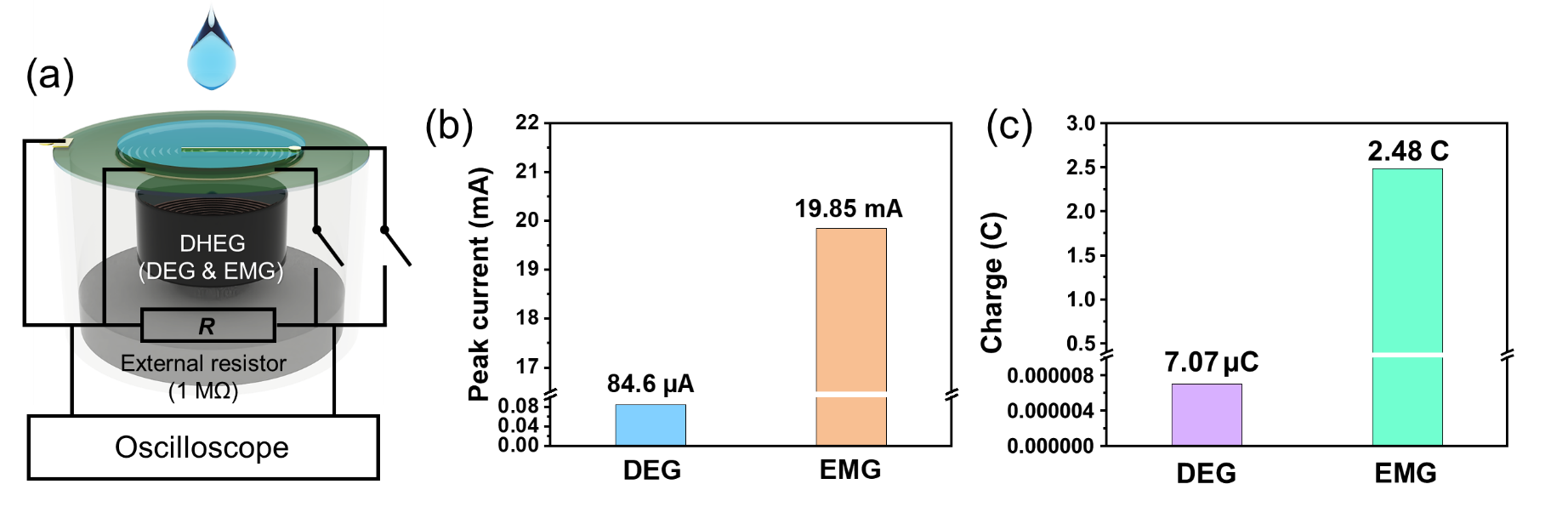


**Figure S5.** a) Voltage measurement circuit for DHEG. b) The output peak currents and c) charges generated from a single droplet impacting on the DEG part and the EMG part, respectively.


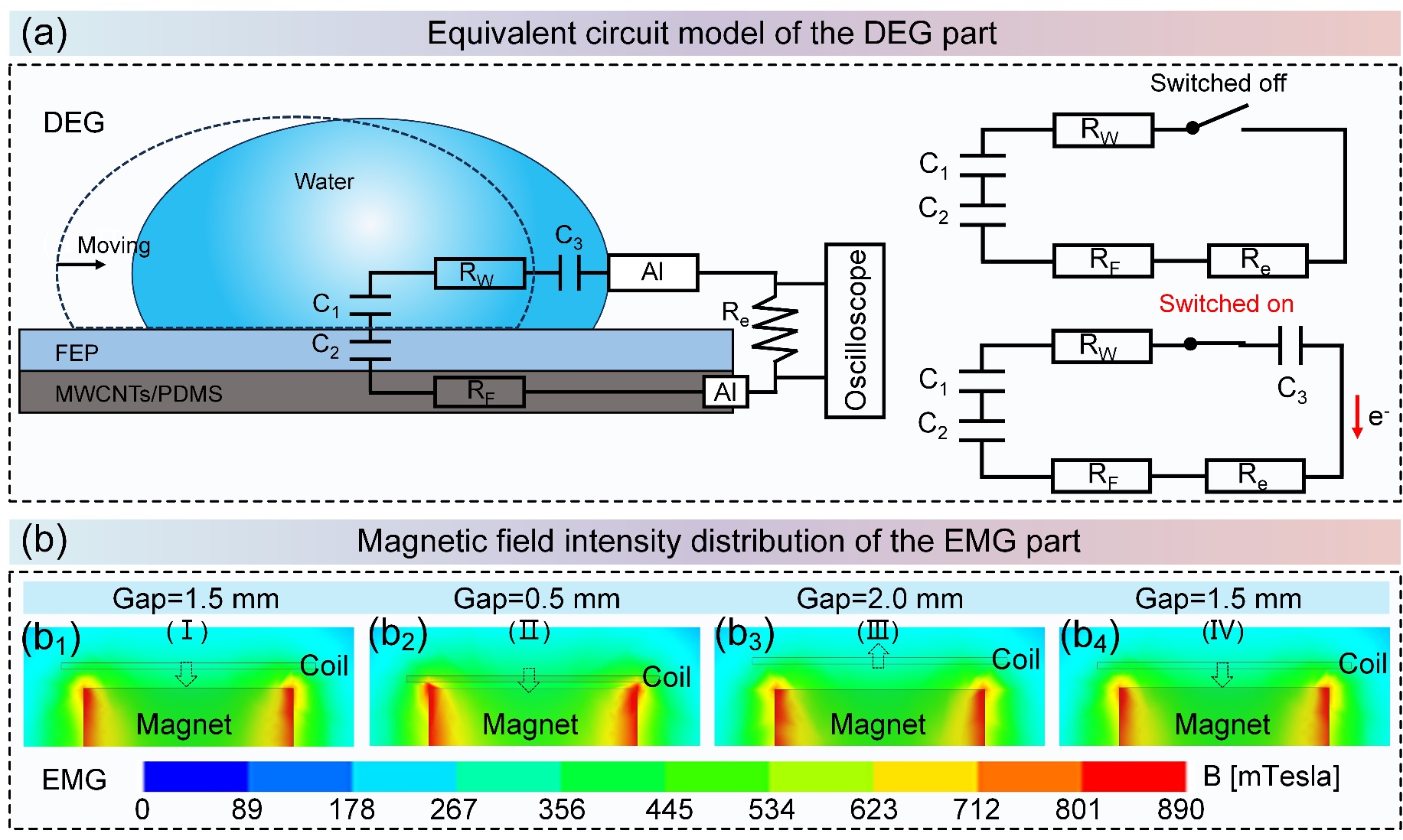


**Figure S6.** a) The equivalent circuit model of the DEG part. Only in the connected state, *C*_3_ presents in the closed circuit. *C*_1_, *C*_2_ and *C*_3_ are the capacitor of the electric double layer between the water and the dielectric, the dielectric/bottom electrode interface and the water/upper electrode interface, respectively. *R*_w_, *R*_E_ and *R*_F_ are the impedance of the water droplet, the external load and the surface of the MWCNTs/PDMS film, respectively. b) The magnetic field intensity distributions of the EMG when subjected to the impact of a single water droplet.


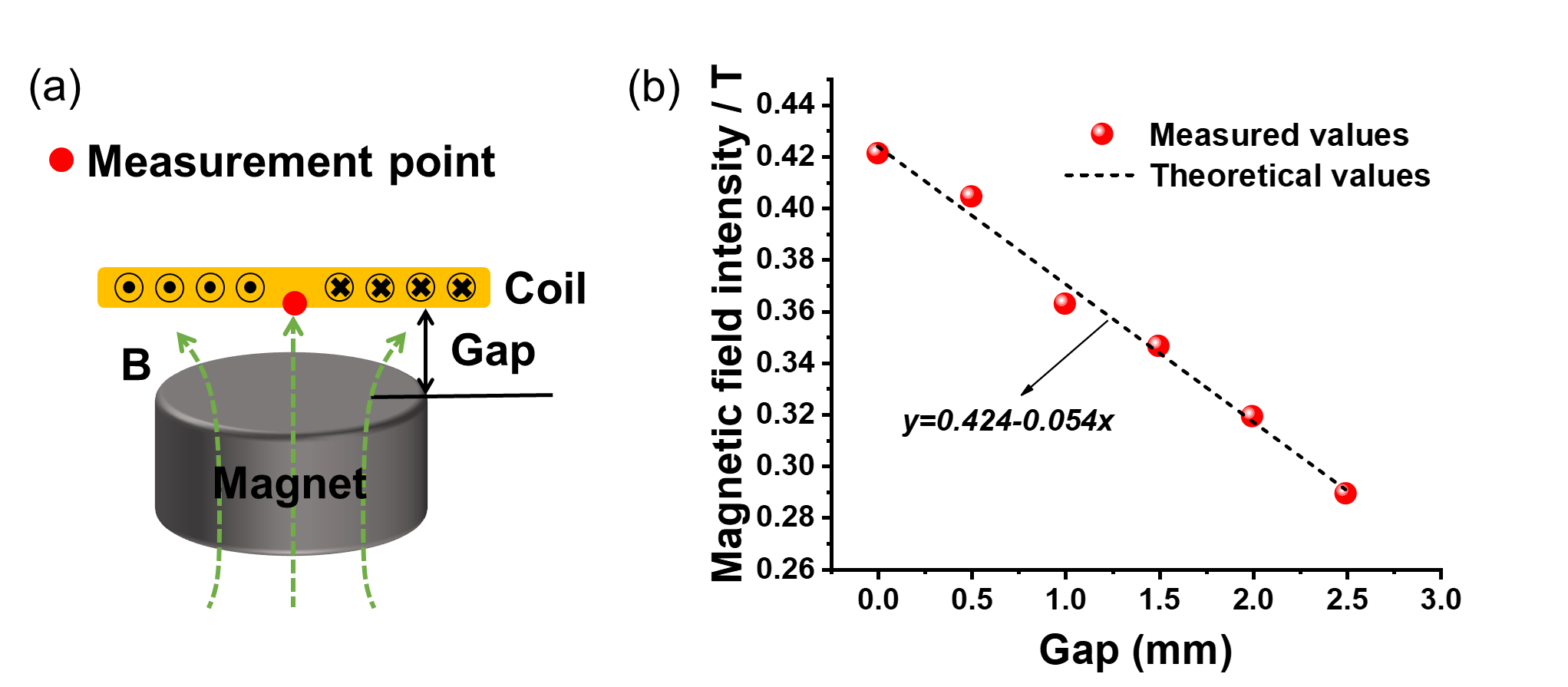


**Figure S7.** a) Magnetic field strength (B) distribution of the device and the corresponding measurement point. b) Curve of magnetic field intensity vs. gap.


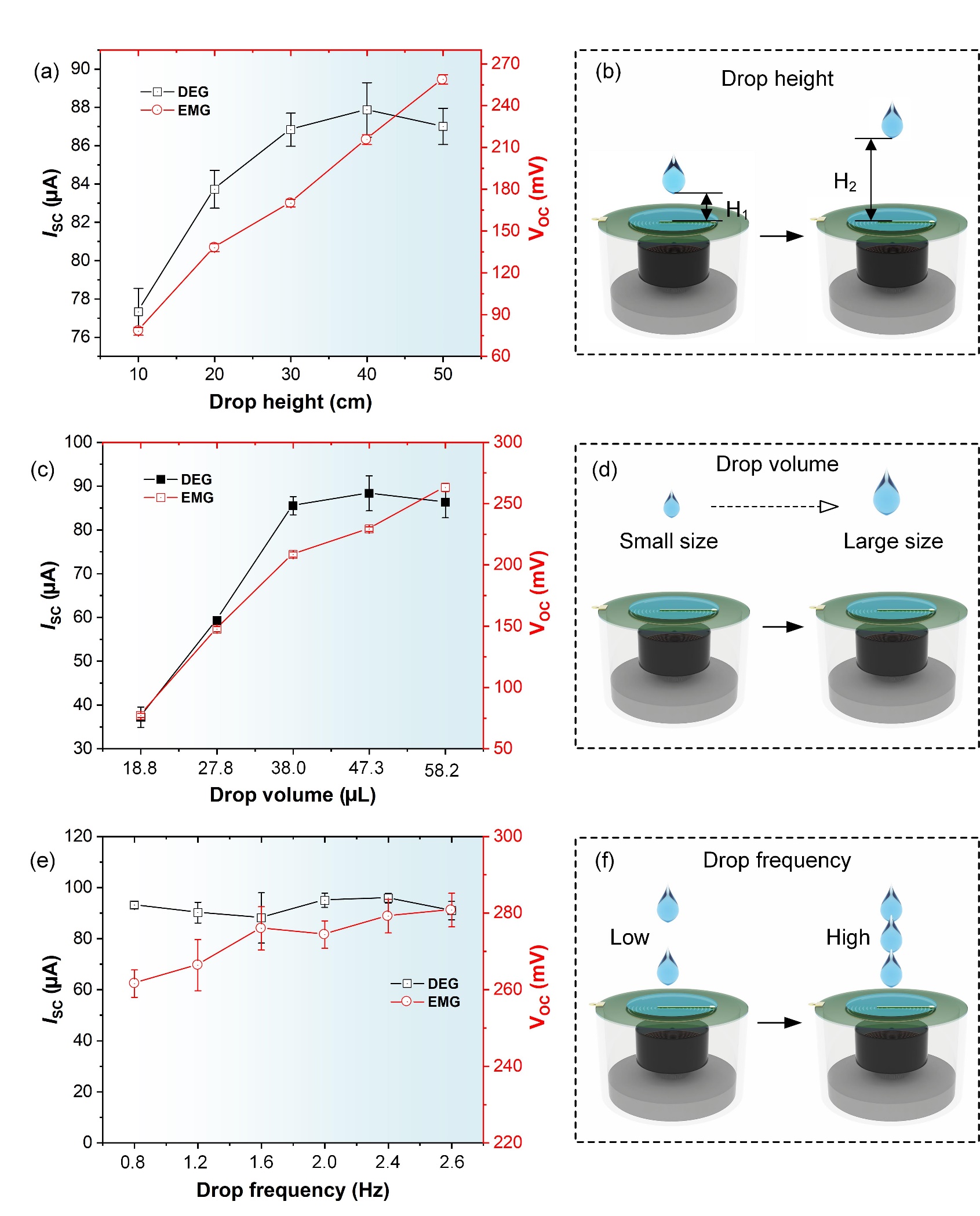


**Figure S8.** Effect of raindrop parameters on electricity produced by the DHEG. a) *I*_SC_ and *V*_OC_ generated by the DEG and EMG parts, respectively, at different drop heights. b) Schematic illustration of different drop heights impacting the DHEG. c) *I*_SC_ and *V*_OC_ generated by the DEG and EMG parts, respectively, at different drop volumes. d) Schematic illustration of different drop volumes impacting the DHEG. e) *I*_SC_ and *V*_OC_ generated by the DEG and EMG parts, respectively, at different drop frequencies. f) Schematic illustration of different drop frequencies impacting the DHEG. Here, the drop volume, height, frequency and tilt angle used in the test were 58.2 µL, 50 cm, 2 Hz and 30°, respectively, except where noted.


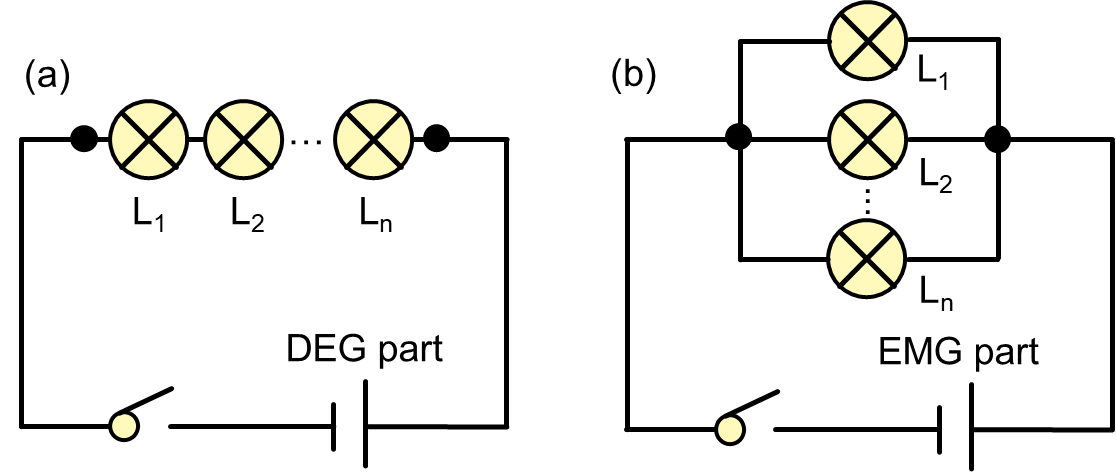


**Figure S9.** (a) Circuit for DEG to light up series-connected LEDs. (b) Circuit for voltage-amplified EMG to light up parallel connected LEDs.


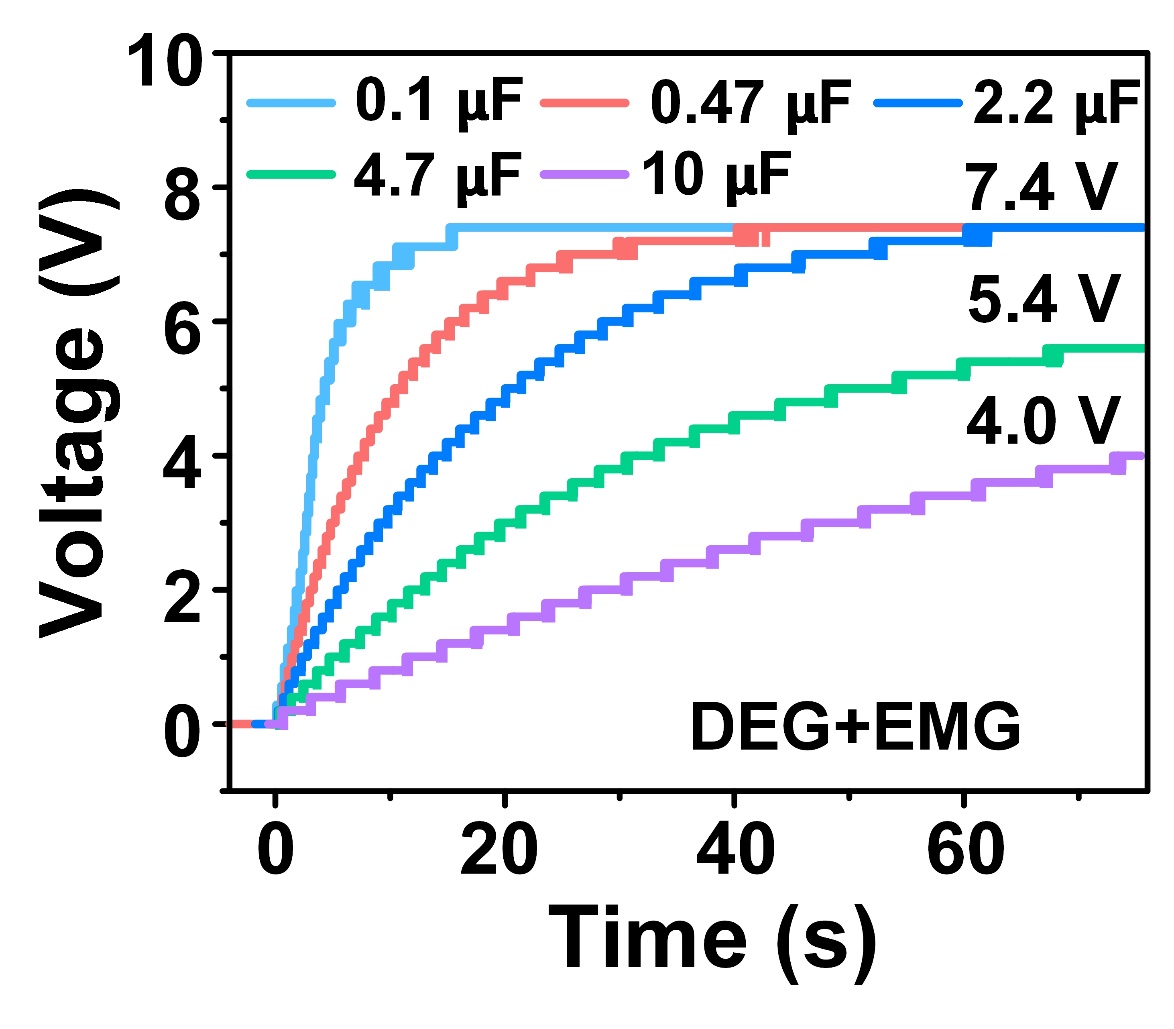


**Figure S10.** Voltage plots of the DHEG (DEG+EMG) for charging different capacitors.


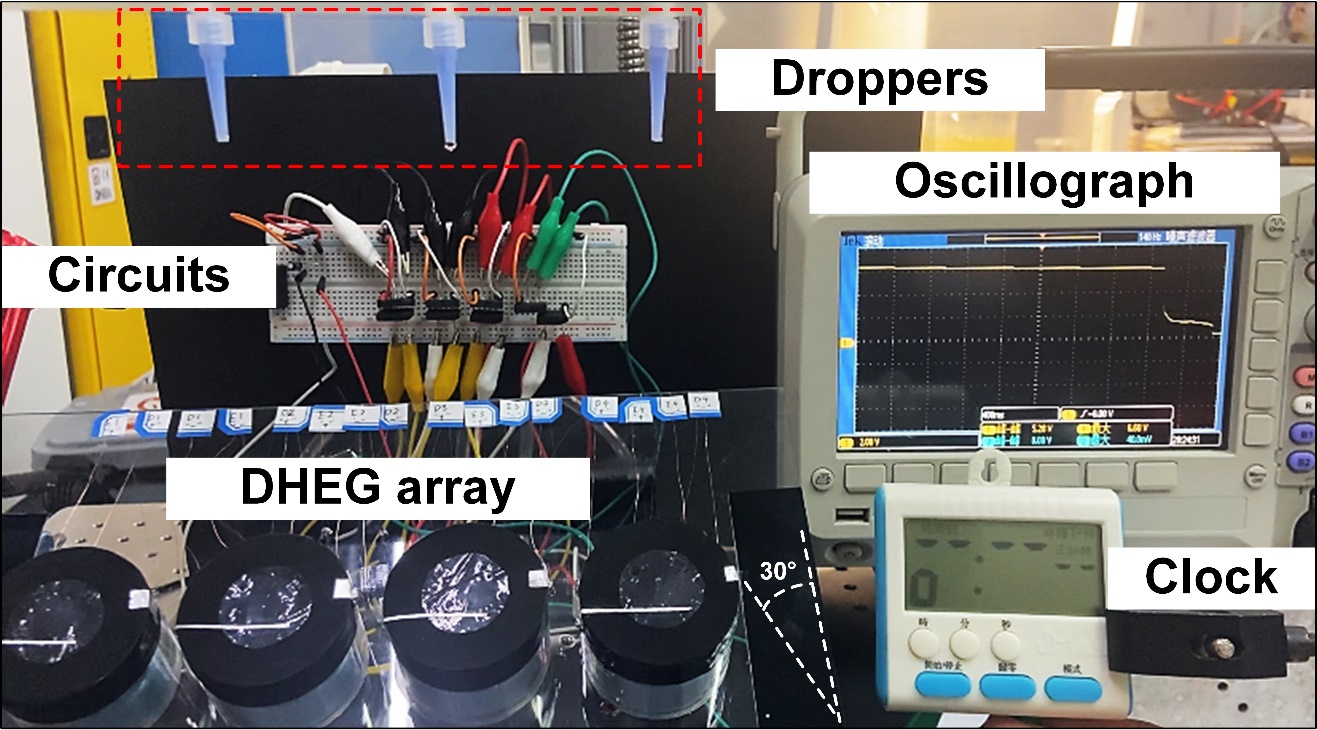


**Figure S11.** Optical image of DHEG array charging capacitor and driving clock.


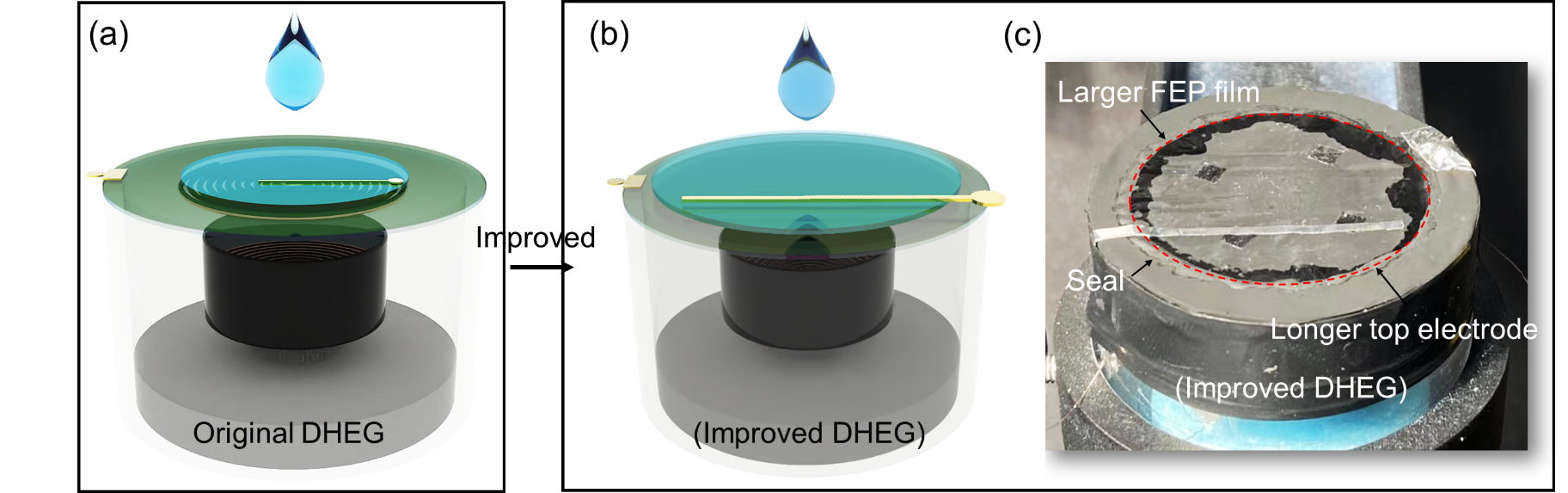


**Figure S12.** A strategy to improve DHEG for collecting more raindrop energy. A prototype of (a) the original DHEG and (b,c) the improved DHEG.


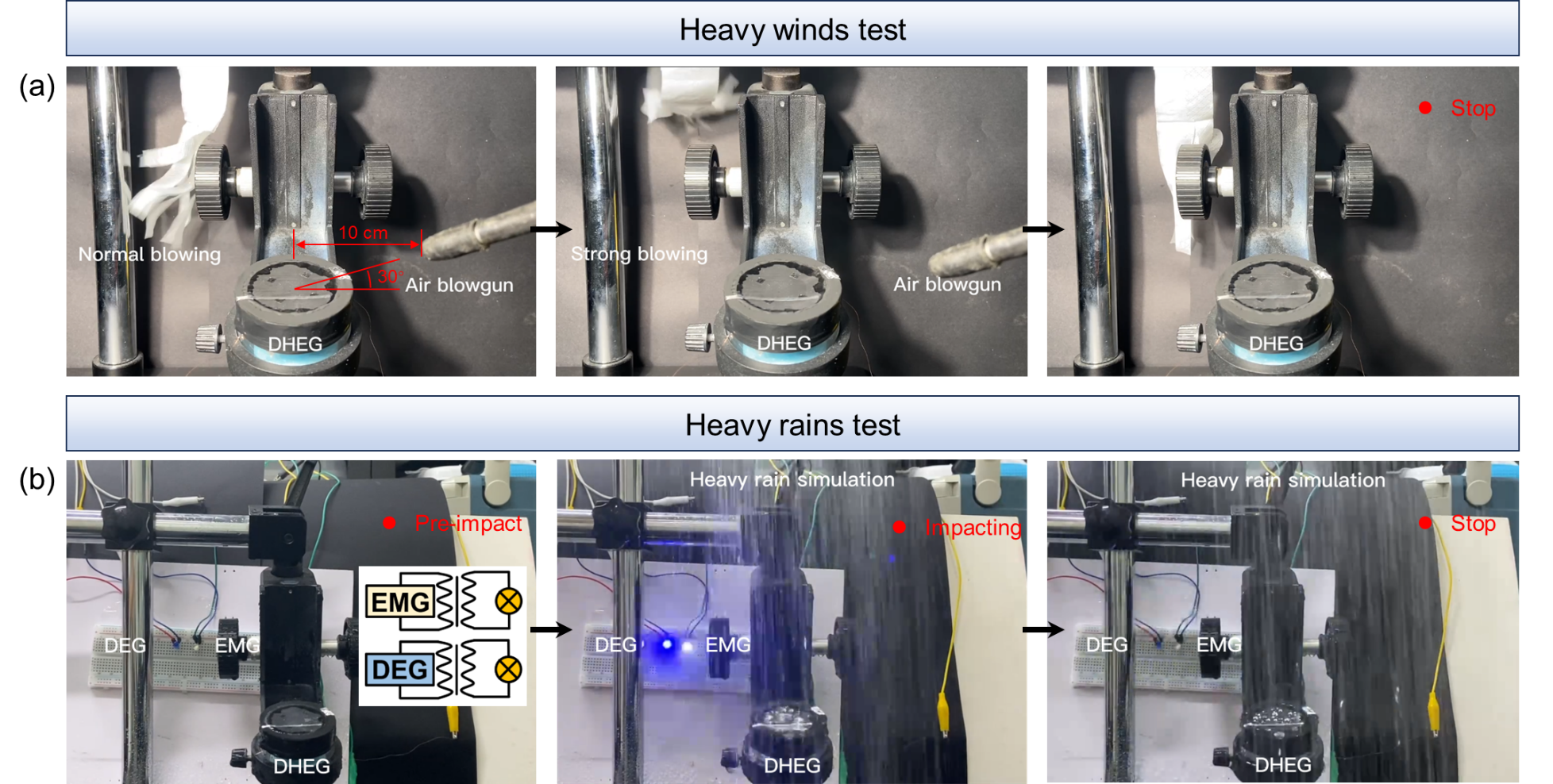


**Figure S13.** The durability of the system in (a) strong wind and (b) heavy rain environments. The DEG and EMG were connected to the amplifier.

**Table S1.** Comparison of the performance of different raindrop-based energy harvesters reported recently.

| **No.** | **Type** | **Voltage** | **Current** | **Water droplet** | **Drop height** | **Power density** | **Charge density** | **Efficiency** | **Year** | **Reference** |
| --- | --- | --- | --- | --- | --- | --- | --- | --- | --- | --- |
| 1 | DEG | ~143.5 V | 270.0 μA | 100.0 μL | 15.0 cm | 50.1 W·m^-2^ | 0.184 mC·m^-2^ | 2.2% | 2020 | [1] |
| 2 | D-TENG | 21.6 V | 0.74 μA | ~60 μL | 2.5 m | 566.2 μW·m^-2^ | 353.86 nC·m^-2^ | - | 2020 | [2] |
| 3 | CTEG | - | 2 mA | 33 µL | 5 cm | 160 W·m^-2^ | 1.8 mC·m^-2^ | 11.8% | 2020 | [3] |
| 4 | SDMS | 19.8 mV | 1.2 mA | 50 µL | 10 cm | 297 mW·m^-2^ | 62.75 mC·m^-2^ | - | 2020 | [4] |
| 5 | SHS-DEG | ~ 200 V | 400 μA | ~4 µL | - | 0.51 W·m^-2^ | - | 1.9% | 2021 | [5] |
| 6 | SMEG | 0.24 V | 1.55 mA | 60 μL | 100 cm | 0.456 W·m^-2^ | 51.5 mC·m^-2^ | - | 2021 | [6] |
| 7 | DC-DEG | ~1600 V | ~400 nA | 80 µL | 15 cm | ~2.67 W·m^-2^ | 0.188 mC·m^-2^ | 2.4% | 2021 | [7] |
| 8 | REH | ~261.2 V | 294.9 μA | 52.8 µL | 1.6 m | 82.66 W·m^-2^ | 134.7 µC·m^-2^ | - | 2021 | [8] |
| 9 | LL-DEG | 184 V | 1.60 mA | 52 µL | 10 cm | 375.6 mW·m^-2^ | 205.8 µC·m^-2^ | 13.7% | 2022 | [9] |
| 10 | DEG | ~150 V | 18 μA | 100 µL | 15 cm | 357 W·m^-2^ | ~44.4 µC·m^-2^ | - | 2022 | [10] |
| 11 | M-DEG | 194.8 V | 40.15 μA | 99.5 μL | 15 cm | - | 90 μC·m^-2^ | - | 2023 | [11] |
| 12 | HDEG | 96 V | 6.86 μA | 38 μL | 60 cm | 1.99 W·m^-2^ | 30.39 μC·m^-2^ | 0.453% | 2023 | [12] |
| 13 | D-TEHG | 3.01 V | 11.2 mA | 58.2 μL | 50 cm | 81.5 mW·m^-2^ | 108.3 mC·m^-2^ | - | 2024 | [14] |
| 14 | D-TENG | 298.27 V | 3.51 mA | 13 mL·min^-1^ | 30 cm | - | 1.28 mC·m^-2^ | - | 2024 | [15] |
| 15 | DHEG | ~86.4 V | ~19.85 mA | 58.2 µL | 50 cm | 1.57 W·m^-2^ | 1631.84 C·m^-2^ | 13.8% | 2024 | [This work] |

**Reference:**

1. W. Xu, H. Zheng, Y. Liu, X. Zhou, C. Zhang, Y. Song, X. Deng, M. Leung, Z. Yang, R. Xu, Z. Wang, X. Zeng, Z. Wang, *Nature* **2020**, *578*, 392-396.
2. S. Nie, H. Guo, Y. Lu, J. Zhuo, J. Mo, Z. Wang, *Adv. Mater. Technol.* **2020**, *5*, 2000454.
3. H. Wu, N. Mendel, S. van Der Ham, L. Shui, G. Zhou, F. Mugele, *Adv. Mater.* **2020**, *32*, 2001699.
4. Z. Ma, J. Ai, Y. Shi, K. Wang, B. Su, *Adv. Mater.* **2020**, *32*, 2006839.
5. L. Wang, Y. Song, W. Xu, W. Li, Y. Jin, S. Gao, S. Yang, C. Wu, S. Wang, Z. Wang, *EcoMat.* **2021**, *3*, e12116.
6. Z. Ma, J. Ai, Y. Yue, K. Wang, B. Su, *Nano Energy* **2021**, *83*, 105846.
7. J. Dong, C. Xu, L. Zhu, X. Zhao, H. Zhou, H. Liu, G. Xu, G. Wang, G. Zhou, Q. Zeng, Q. Song, *Nano Energy* **2021**, *90*, 106567.
8. X. Xu, Y. Wang, P. Li, W. Xu, L. Wei, Z. Wang, Z Yang, *Nano Energy* **2021**, *90*, 106573.
9. D. Yoo, S. Kim, Y. Joung, S. Jang, D. Choi, D. Kim, *Nano Energy* **2022**, *99*, 107361.
10. N. Zhang, H. Zhang, W. Xu, H. Gu, S. Ye, H. Zheng, Y. Song, Z. Wang, X. Zhou, *Droplet* **2022**, *1*, 56-64.
11. K. Wang, W. Xu, J. Li, H. Zheng, S. Sun, W. Song, Y. Song, Z. Ding, R. Zhang, Y. Sun, H. Zhang, Nano Energy **2023**, *111*, 108388.
12. D. Kam, G. Gwon, S. Jang, D. Yoo, S. J. Park, M. La, D. Choi, *Adv. Mater.* **2023**, *35*, 2303681.
13. Y. Zhang, J. Liu, J. Zhang, Y. Chen, Y. Zhou, X. Liu, *Nano Energy* **2024**, *121*, 109253.
14. J. Meng, L. Zhang, H. Liu, W. Sun, W. Wang, H. Wang, D. Yang, M. Feng, Y. Feng, D. Wan, *Adv. Energy Mater.* **2024**, *14*, 2303298.
